# Supplementary material for: Loss of PRC2 subunits primes lineage choice during exit of pluripotency
Source: Nat Commun. 2021 Nov 30;12:6985. doi: 10.1038/s41467-021-27314-4 (PMC8632979; doi:10.1038/s41467-021-27314-4)
Supplement: Supplementary file 1 — Supplemental Information [file 41467_2021_27314_MOESM1_ESM.pdf]

Supplemental Information

Loss of PRC2 subunits primes lineage choice during exit of pluripotency

Chet H Loh[1], Siebe van Genesen[1], Matteo Perino[1,2], Magnus R Bark[1], and Gert Jan C Veenstra[1*].

[1] Department of Molecular Developmental Biology, Faculty of Science, Radboud Institute for Molecular Life Sciences, Radboud University, Nijmegen, The Netherlands.
[2] Present address: Genome Biology Unit, European Molecular Biology Laboratory (EMBL), Heidelberg, Germany

[*] email: g.veenstra@science.ru.nl

Supplemental Figure 1

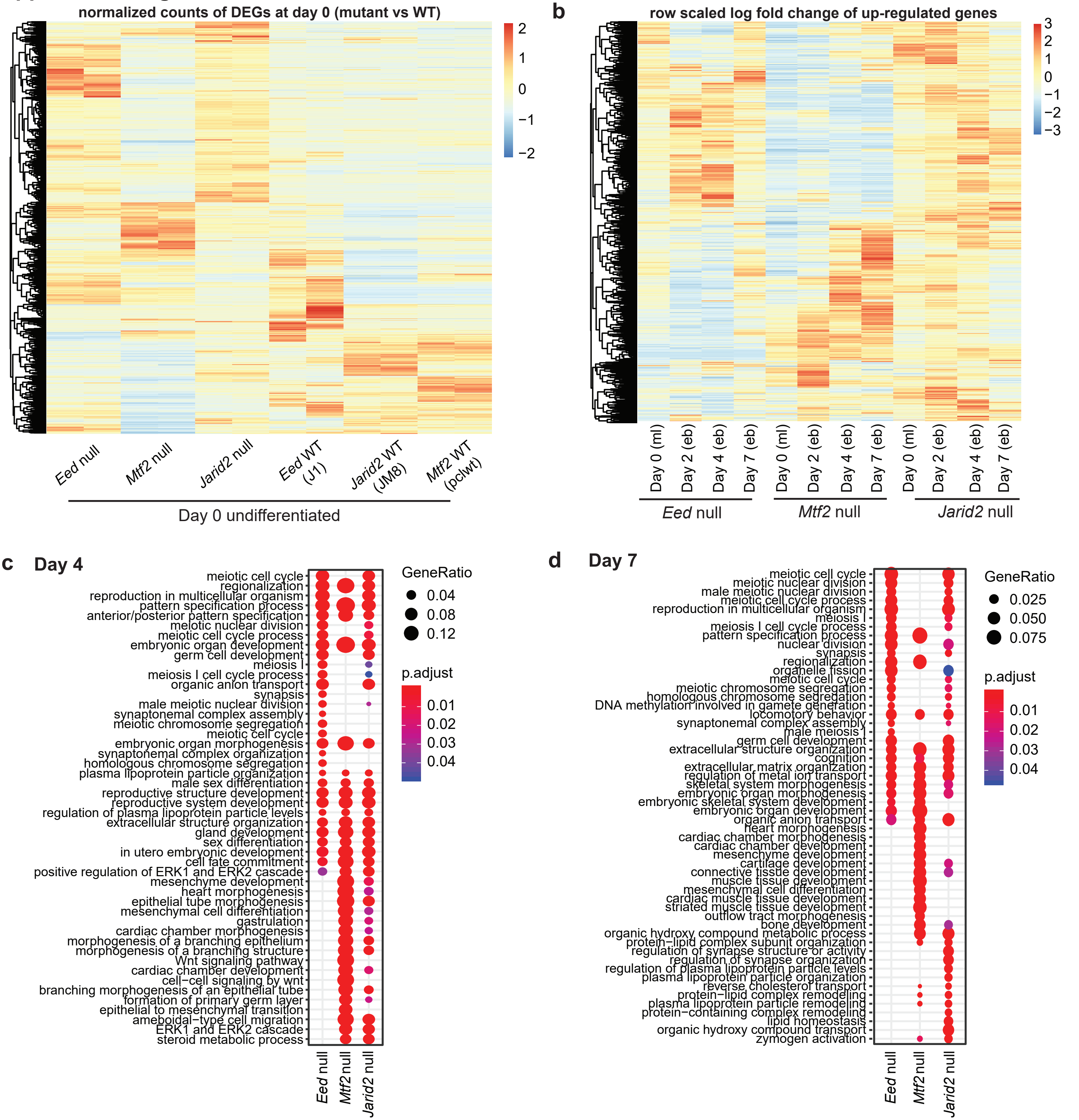

Supplemental Fig.1 Bulk-RNA seq validation of PRC2 mutant phenotypes using matched WT. a. Heatmap of differentially expressed genes (DEG) at day 0 undifferentiated state in different mutants and WTs (normalized counts). **b.** Heatmap of upregulated genes in mutants against their WTs (row-scaled z-scores). ml, monolayer; eb,embryoid body. **c-d.** Gene ontology plots of terms associated with mutants at respectively day 4 and day 7 based on differentially upregulated genes compared to matched WT cells. Hypergeometrical testing (two-sided) were perfomed and p-values were adjusted for multiple comparison, and q-values were also calculated for FDR control.

Supplemental Figure 2

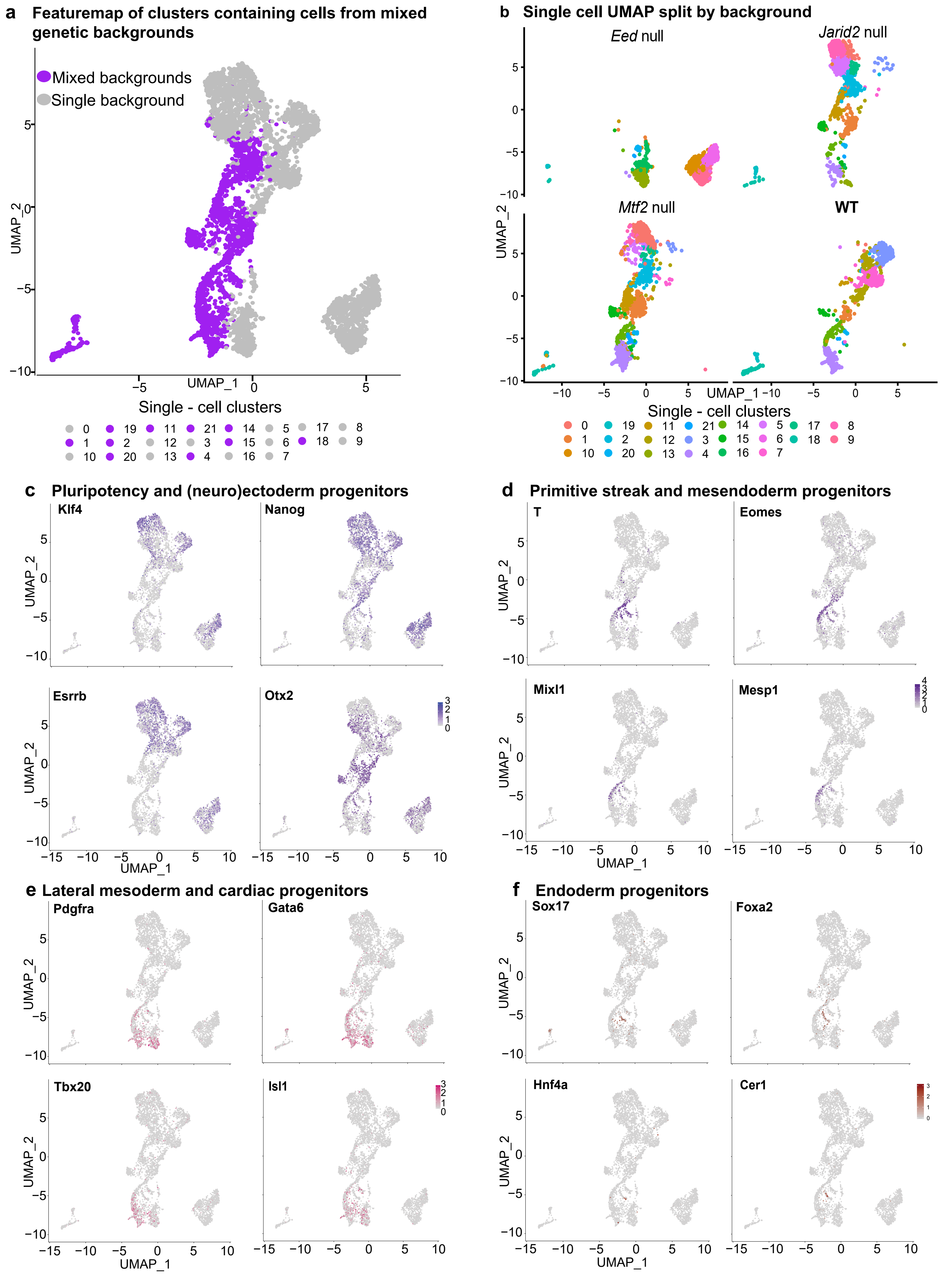

Supplemental Fig.2. Gene expression in wild type and mutant embryoidd bodies at single-cell level. a, Feature map showing the clusters which contain mix genetic background (purple) and others which contain single background. A mixed genotype cluster is defined as a cluster where at most 80% of the cells share the same genotype. **b**, single cell umap clusters, split by different genetic background. **c - f**, Feature maps depicting the expression pattern and levels of selected key lineage genes from different germ layers during development.

Supplemental Figure 3

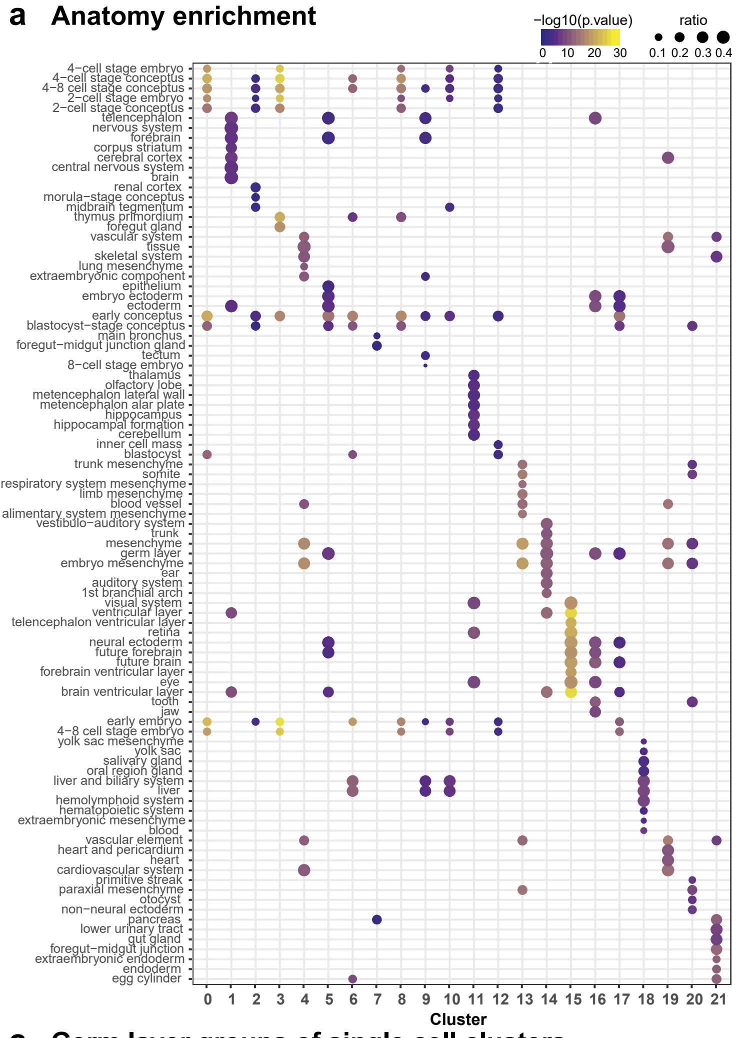

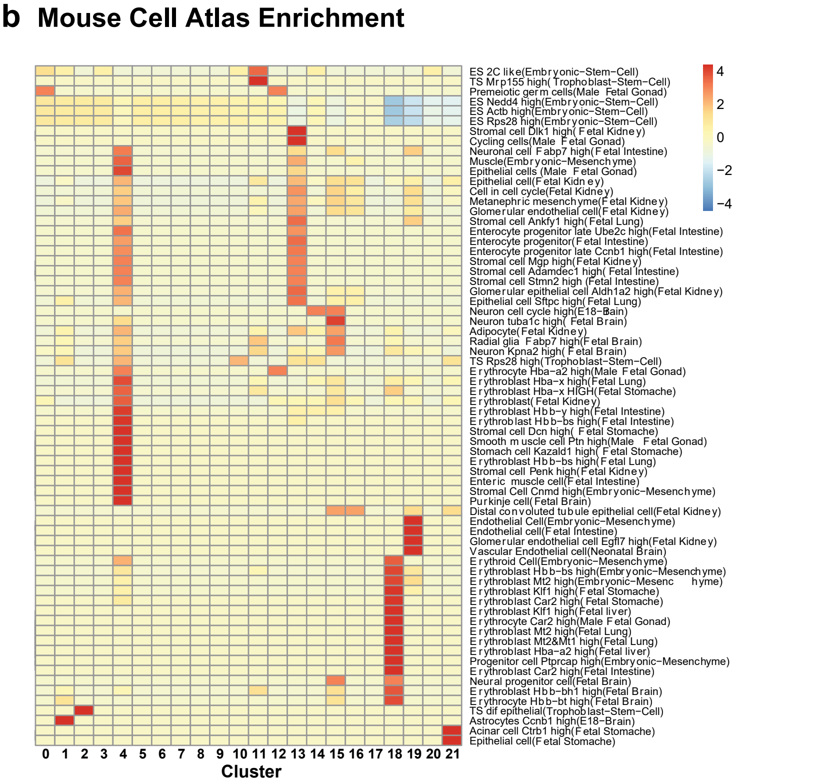

c Germ layer groups of single cell clusters

Clusters	Mouse Anatomy Ontology	Broad Germ Layer Category
4	Cardiovascular System; Vascular Element	Mesodermal
13	Somites;Blood Vessels	
19	Heart; Heart and Pericardium	
21	Gut; Foregut - Midgut Junction; Endoderm	Endodermal
1	Cerebral Cortex; Ectoderm	Ectodermal
11	Eye, Retina, Visual system	
15	Brain Ventricular Area Future Forebrain	
16	Future forebrain, ectoderm	

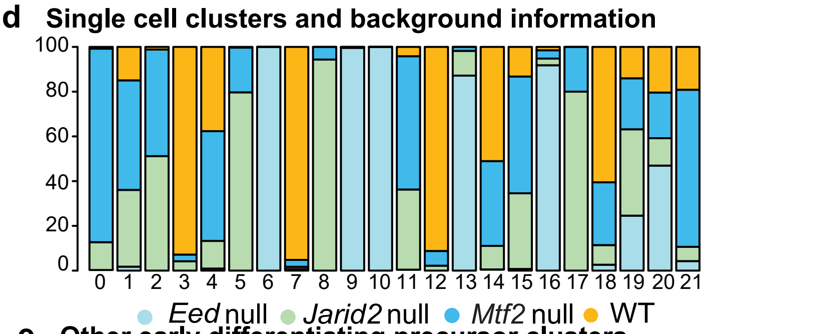

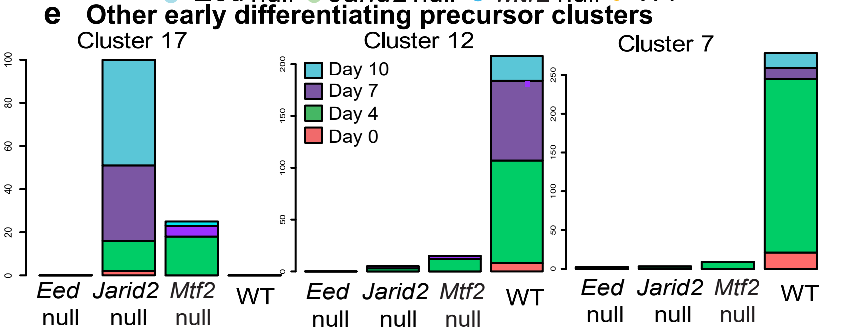

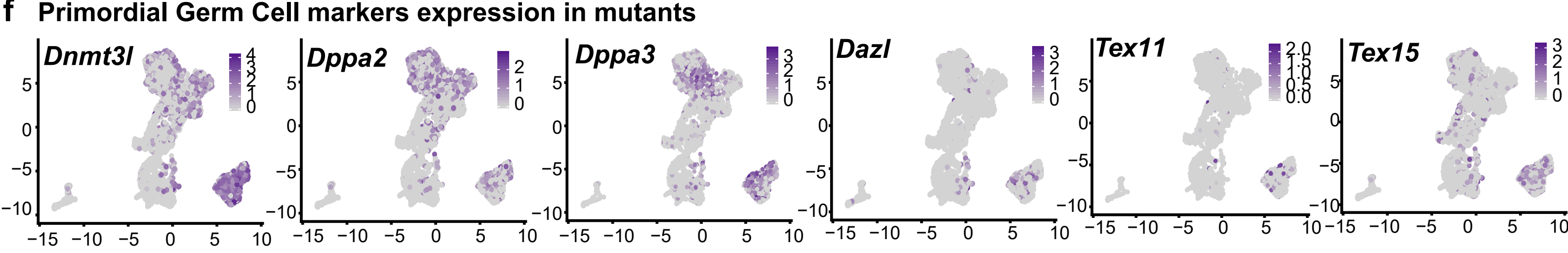

Supplemental Fig.3 Cell clusters in embryoid bodies in Polycomb mutant ESCs a, Dotplot of enriched Anatomical terms representing different clusters from single-cell analyses. **b**, Heatmap of enrichment of single-cell clusters compared to clusters from the Mouse Cell Atlas database. **c**, Table of annotated clusters based on Anatomy ontology and broad germ layer classification. **d**, Barplot showing the proportions of cells from different genetic backgrounds (colours) in all single-cell clusters. **e**, Barplots of number of cells from different backgrounds in other early differentiating precursor clusters from single-cell data apart from cluster 5 shown in main panel. **f**, featuremaps showing relative expression of selected primordial germ cell markers during embryonic development.

Supplemental Figure 4

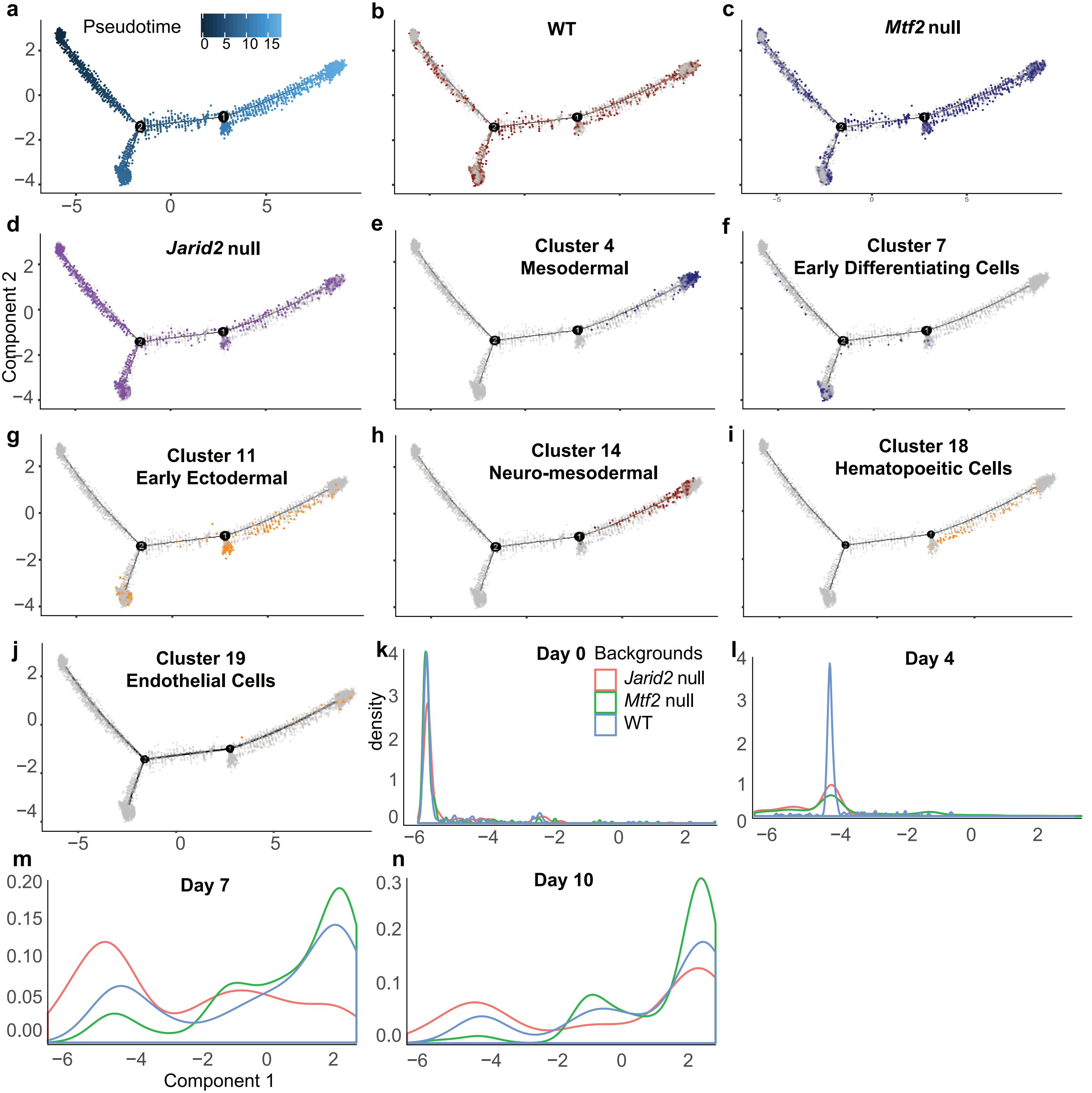

Supplemental Fig.4 Lineage trajectory analysis confirmed faster generation of cell types in *Mtf*2 null cells. a, pseudotime of individual cells. **b - d,** pseudotime plots overlaid with different genetic backgrounds. **e - j,** selected single cell clusters from different timepoints. **k - n,** different density plots of number of cells from different genetic backgrounds over pseudotime (x-axis)

Supplemental Figure 5

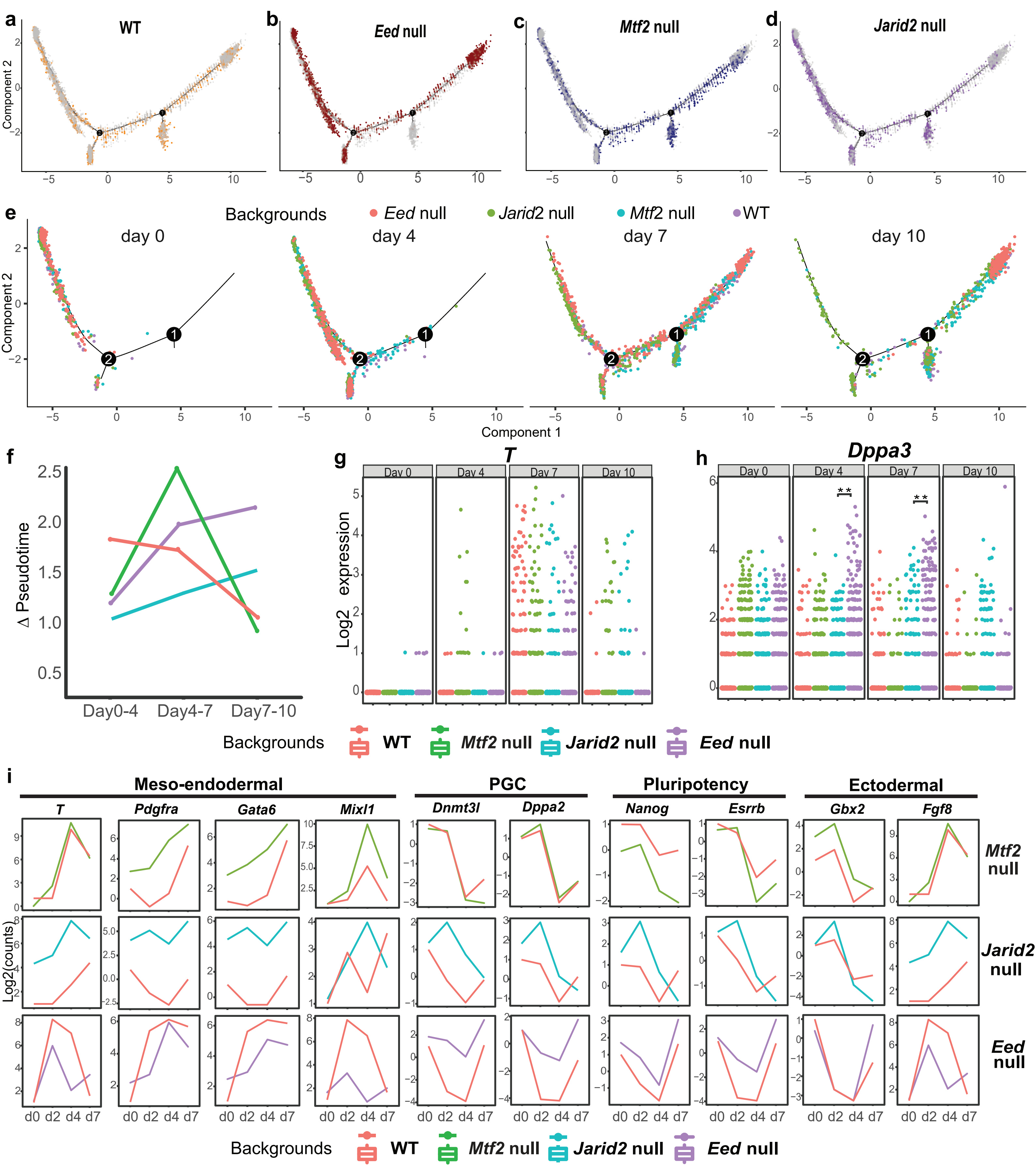

Supplemental Fig.5 Single cell trajectory analyses including Eed null cells depicting **a - d,** pseudotime plots overlaid with different genetic backgrounds, cells ordered by hyper variable gene list, **e,** pseudotime plots for different backgrounds across split by timepoint. **f,** Delta pseudotime plot(speed of differentiation) for all samples including *Eed* null cells. **g - h,** expression dynamics of selected keymarkers during differentiation. double asterisks (*) represents a Wilcoxon signed-rank test p-value < 1 X 10-4, **i,** expression of keydifferentiation markers comparing mutant versus respective wild-type cells across timepoints (normalized over d0 of WT)

Supplemental Figure 6

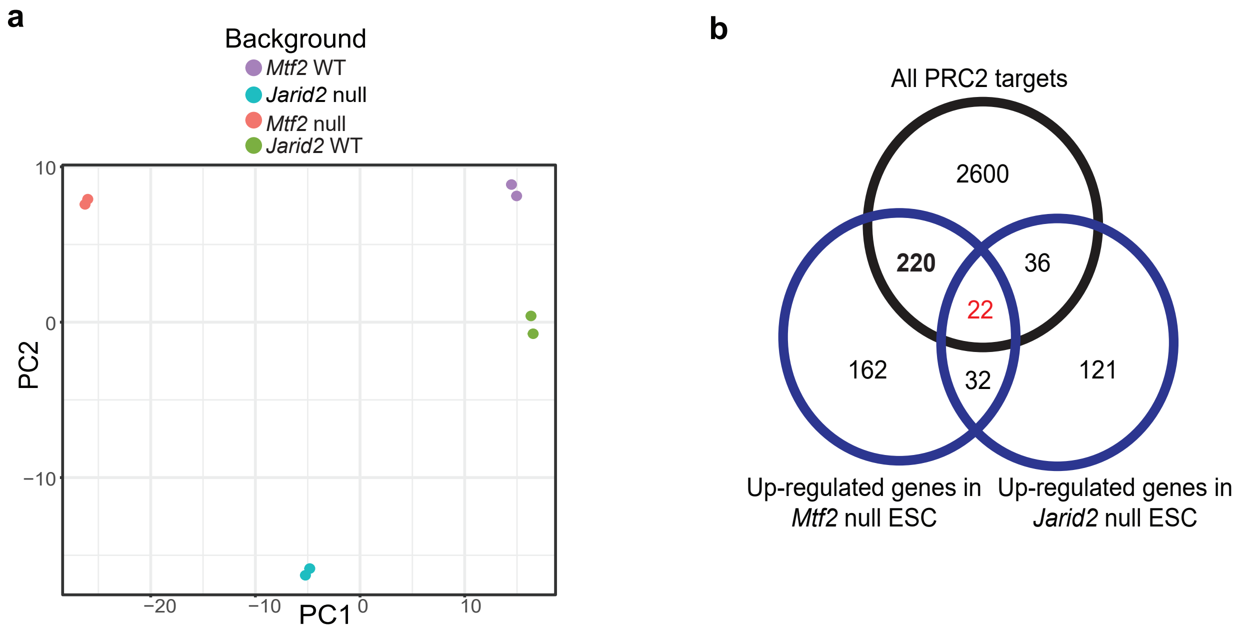

Supplemental Fig.6 Depression of PRC2 targets in *Mtf*2 and *Jarid*2 null ES cells at undifferentiated state. a, PCA analysis showing variance between background during undifferentiated state. b, Venn diagramm for overlapping up-regulated genes between *Mtf2* and *Jarid2* null, that are also PRC2 targets.

Supplemental Figure 7

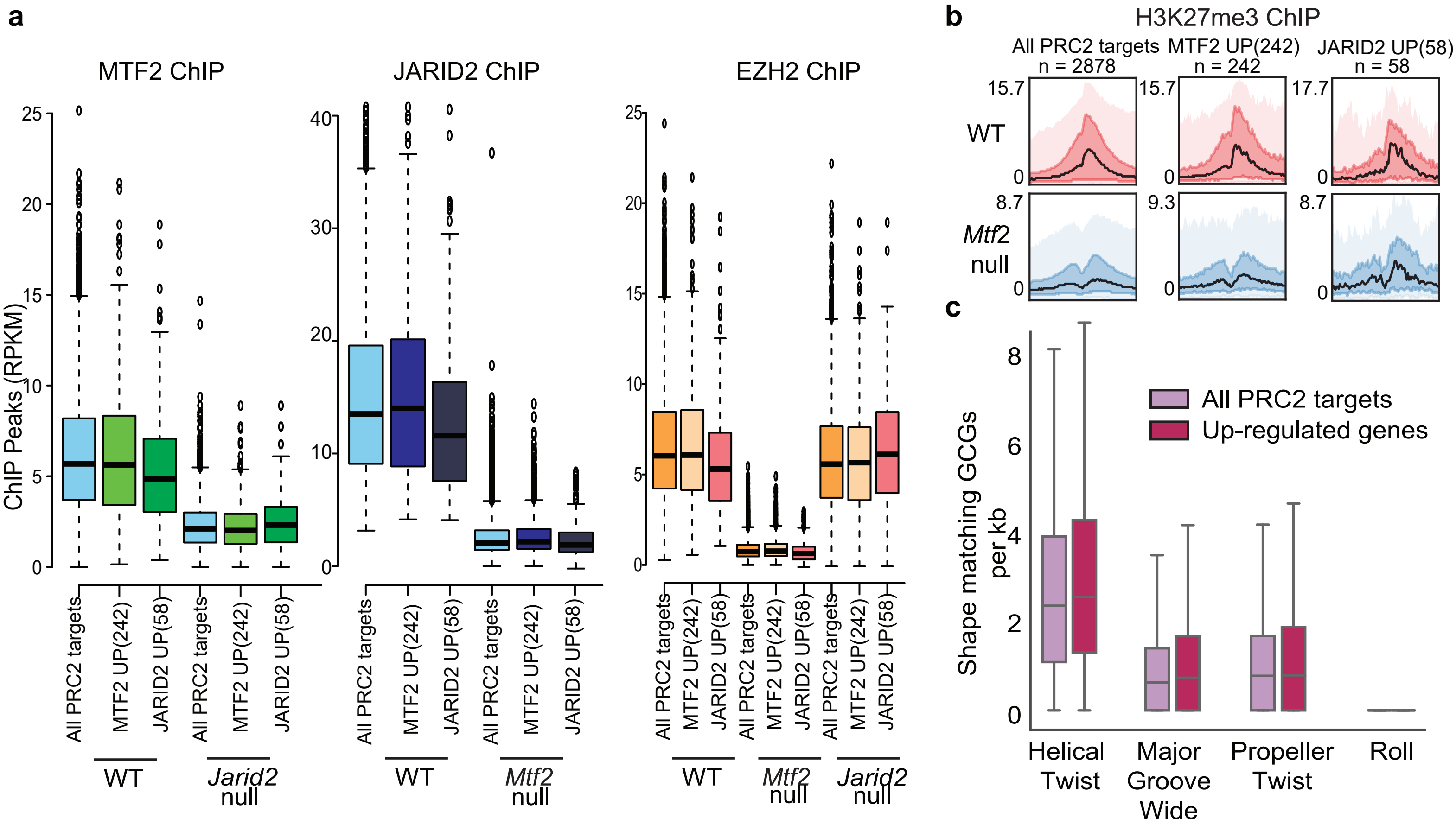

Supplemental Fig.7 Key PRC2-repressed lineage transcription factors are poised for activation. a, Barplots showing the ChIP RPKMs for MTF2, JARID2 and EZH2 comparing between all PRC2 targets and the upregulated genes in *Mtf2* and *Jarid2* null cells. n = 2878 for all PRC2 targets, n = 242 for upregulated Mtf2 null genes and n = 58 for upregulated Jarid2 null genes. Whisker ends of boxplot represents the maximum (top) and minimum values respectively. Top and bottom of boxplots represent 75th and 25th percentile values respectively and finally, median values are shown as black lines within the boxplots. **b,** Bandplots depicting the averaged profiles of peaks (RPKM) in a +/- 5kb region around the TSS of genes in either 1) All PRC2 targets (left), 2) All up-regulated PRC2 targets in *Mtf2* null (middle) and 3) All up-regulated PRC2 targets in *Jarid2* null cells (right). Values shown are normalized (RPKM). The median enrichment is visualized as a black line with the 50th and 90th percentile as a dark and light colour respectively. **c,** Number of shape matching CpG islands in the promoters of all PRC2 targets and the upregulated genes in *Mtf2* null cells. n = 2878 for All PRC2 targets and n = 242 for Up-regulated genes. Whisker ends of boxplot represents the maximum (top) and minimum values respectively. Top and bottom of boxplots represent 75th and 25th percentile values respectively and finally, median values are shown as gray lines within the boxplots.

Supplemental Figure 8

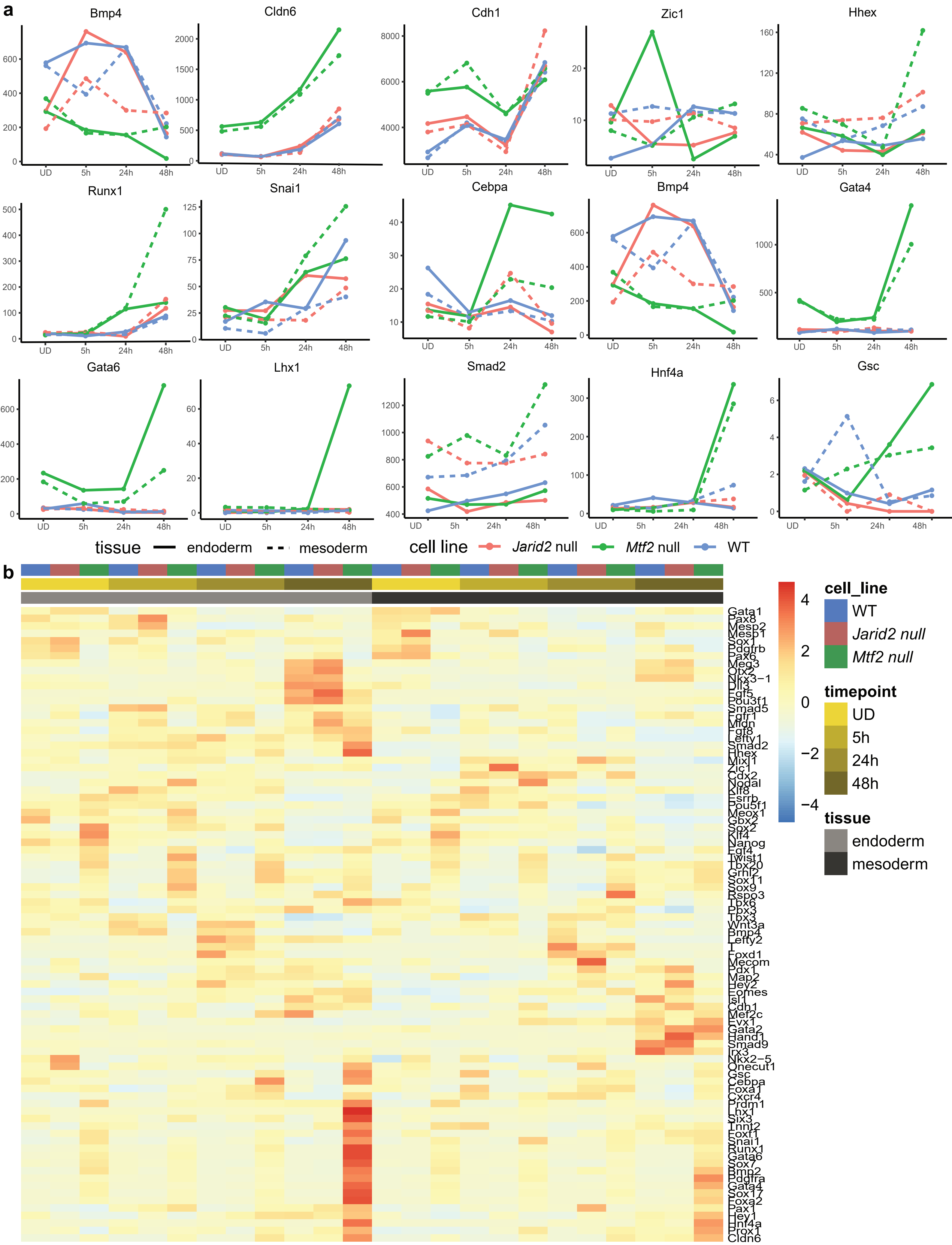

Supplemental Fig.8 Directed differentiation of Mtf2 and Jarid2 null cells. a, Lineplots showing the expression of key early developmental factors (normalized counts from bulk-RNA seq) **b**, Heatmap depicting the expression differences of key mesodermal and endodermal gene markers between different differentiation experiments(mesoderma and endoderm) and across different timepoints of differentiation.

Supplemental Figure 9

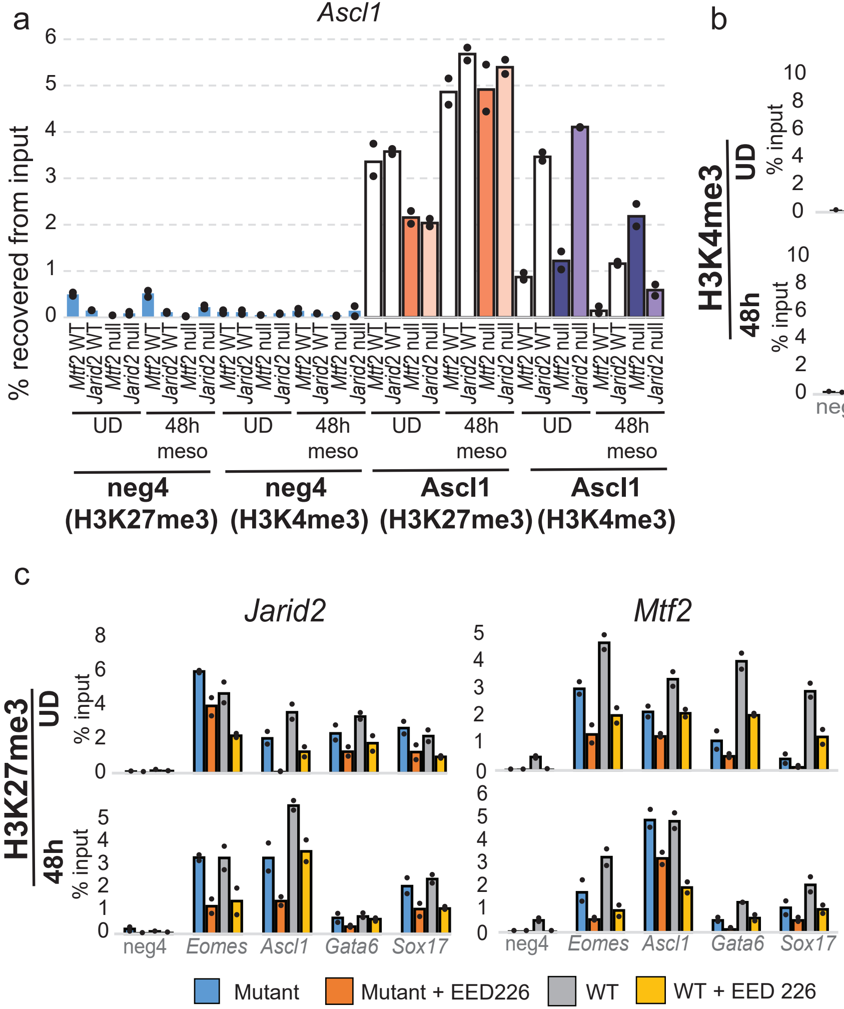

Supplemental Fig. 9 H3K4me3 and H3K27me3 levels in *Mtf2* null cells during mesoderm differentiation.
a, Barplot of % input recovered for H3K27me3 and H3K4me3 ChIP for *Ascl1* done on qPCR. Eachbar represents an average of the % input from duplicates, shown in dots. **b,** Barplots showing % of input for activating H3K4me3 ChIP - qPCR performed with additional control of adding chemical inhibitor EED226. Each bar is an average of duplicates from the experiment. **c,**Barplots showing % if input recovered from repressive mark H3K27me3 ChIP qPCR during differentiation and with addition of EED226 inhibitor.

Supplemental Table 1. Germ layer enrichment analyses

Cell number in clusters

	Cluster	WT	Mtf2 null	Jarid2 null	Eed null
**Mesodermal**	**4**	122	159	40	3
	**19**	8	13	22	14
	**13**	0	4	25	197
	**SUM**	130	176	87	214
**Endodermal**	**21**	9	33	3	2
	**SUM**	9	33	3	2
**Ectodermal**	**11**	10	143	86	1
	**15**	18	71	46	1
	**1**	51	167	117	6
	**16**	2	5	4	123
	**SUM**	81	386	253	131
**Early Differentiating**	**5**	1	63	251	0
	**17**	0	25	100	0
	**12**	208	15	5	0
	**7**	278	9	3	2
	**SUM**	487	112	359	2
**ES and other**	**0, 2, 3, 6, 8, 9, 10, 14,18,20**				
	**SUM**	494	636	522	832

Cell numbers for each genotype per timepoint

	WT	Mtf2 null	Jarid2 null	Eed null
**Totals**	1201	1343	1224	1181
**Day 0**	340	347	337	340
**Day 4**	331	331	321	320
**Day 7**	316	344	302	319
**Day 10**	214	321	264	202

Summary table	WT	Mtf2 null	Jarid2 null	Eed null
**Totals**	1201	1343	1224	1181
**Mesoderm**	130	176	87	214
Fold change against WT	1.00	1.21	0.66	1.67
2Log fold change	0.00	0.28	-0.61	0.74
**Endoderm**	9	33	3	2
Fold change against WT	1.00	3.28	0.33	0.23
2Log fold change	0.00	1.71	-1.61	-2.15
**Ectoderm**	81	386	253	131
Fold change against WT	1.00	4.26	3.06	1.64
2Log fold change	0.00	2.09	1.62	0.72
**Others (Incl. early precursors)**	981	748	881	834
Fold change against WT	1.00	0.68	0.88	0.86
2Log fold change	0.00	-0.55	-0.18	-0.21
**Early Differentiating Precursors**	487	112	359	2
Fold change against WT	1.00	0.21	0.72	0.00
2Log fold change	0.00	-2.28	-0.47	-7.90
