# Supplementary material for: Loss of PRC2 subunits primes lineage choice during exit of pluripotency
Source: Nat Commun. 2021 Nov 30;12:6985. doi: 10.1038/s41467-021-27314-4 (PMC8632979; doi:10.1038/s41467-021-27314-4)
Supplement: Supplementary file 3 — Description of additional Supplementary File [file 41467_2021_27314_MOESM3_ESM.pdf]

Description of Additional Supplementary data files

File Name: Supplemental Data 1
Description: Table of PRC2 target genes in mouse embryonic stem cells, as defined by EZH2 binding (ChIP)
File Name: Supplemental Data 2
Description: Gimme Maelstrom derived table of transcription factor motifs that are found to be enriched in the promoter region of differentially expressed genes in WT, Mtf2 and Jarid2 null cells at pluripotent stage. Pclwt and Jm8 refer to the genetically matched wild-type cells of Mtf2 and Jarid2 null cells, respectively.

File Name: Supplemental Data 3
Description: Table containing the validation of predicted transcription factor motifs (from Gimme Maelstrom) against public motif databases such as JASPAR, SELEX and ChIP-seq datasets.
