# Supplementary material for: Loss of PRC2 subunits primes lineage choice during exit of pluripotency
Source: Nat Commun. 2021 Nov 30;12:6985. doi: 10.1038/s41467-021-27314-4 (PMC8632979; doi:10.1038/s41467-021-27314-4)
Supplement: Supplementary file 7 — Reporting Summary [file 41467_2021_27314_MOESM7_ESM.pdf]

nature research

Corresponding author(s): Gert Jan Veenstra

Last updated by author(s): 22-10-2021

Reporting Summary

Nature Research wishes to improve the reproducibility of the work that we publish. This form provides structure for consistency and transparency in reporting. For further information on Nature Research policies, see our Editorial Policies and the Editorial Policy Checklist.

Statistics

For all statistical analyses, confirm that the following items are present in the figure legend, table legend, main text, or Methods section.

n/a	Confirmed	
☐	☒	The exact sample size (*n*) for each experimental group/condition, given as a discrete number and unit of measurement
☐	☒	A statement on whether measurements were taken from distinct samples or whether the same sample was measured repeatedly
☐	☒	The statistical test(s) used AND whether they are one- or two-sided<br>*Only common tests should be described solely by name; describe more complex techniques in the Methods section.*
☒	☐	A description of all covariates tested
☐	☒	A description of any assumptions or corrections, such as tests of normality and adjustment for multiple comparisons
☒	☐	A full description of the statistical parameters including central tendency (e.g. means) or other basic estimates (e.g. regression coefficient) AND variation (e.g. standard deviation) or associated estimates of uncertainty (e.g. confidence intervals)
☐	☒	For null hypothesis testing, the test statistic (e.g. *F*, *t*, *r*) with confidence intervals, effect sizes, degrees of freedom and *P* value noted<br>*Give P values as exact values whenever suitable.*
☒	☐	For Bayesian analysis, information on the choice of priors and Markov chain Monte Carlo settings
☒	☐	For hierarchical and complex designs, identification of the appropriate level for tests and full reporting of outcomes
☒	☐	Estimates of effect sizes (e.g. Cohen's *d*, Pearson's *r*), indicating how they were calculated

Our web collection on statistics for biologists contains articles on many of the points above.

Software and code

Policy information about availability of computer code

Data collection	No software was used to collect data
Data analysis	Sequencing libraries were sequenced on the NextSeq 500 (Illumina).<br>Single – cell RNA:<br>Raw reads were mapped and aligned to the mouse genome GRCm38/mm10 database using the Bowtie266 alignment tool. Aligned reads were indexed and the final count table was derived using HTseq67. R package 'Scater'68 was used for sample filtering and quality check, to remove dropouts (cells with < 5 reads) and cells with too few recovered genes (<500). The data was than analysed using R package 'Seurat'69 (v3) for batch, read counts and gene counts normalization. In summary, after filtering, we captured the transcriptomes of 1196 WT cells, 1254 Mtf2 null cells, 1196 Jarid2 null cells and 811 Eed null cells. After filtering, technical confounders such as total number of counts and features were also normalized using the 'LogNormalize' function with a scale factor of 10,000. Next, feature selection was performed using the 'vst' method in Seurat to identify the top 2000 most hypervariable genes (HVGs). Then, the dataset was batch corrected using the linear regression model in built in Seurat to regress out unwanted technical effects of libraries (batches) via scaling. Thereafter, the dataset was subjected to linear dimensional reduction using a Principal Compenent Analysis of the top 15 principal components (PCs), determined by an elbow plot of the PCs. The cells are then clustered using a shared nearest neighbor (SNN) modularity optimization - based clustering algorithm at a resolution of 2.5 and the 22 different clusters were projected onto a 2-D UMAP for data visualization. Time course trajectory analyses were performed using the package 'Monocle v2'70. The clusters information from Seurat were imported into Monocle v2 for analyses and the top 2000 HVG detected in Seurat were used to order the cells during pseudotime analyses. All cells were used in combination for the trajectory analyses and no ground states were set for the pseudotime analysis. Actual timepoint information and cluster information were overlaid onto the trajectory plots. Cluster identities were defined by Anatomy ontology71 and correlated with Mouse Cell Atlas33 (MCA) cluster data. The single cell data can be downloaded and viewed in a user interface via the following repository - https://github.com/chethloh/PRC2_singlecelldata<br><br>Bulk – RNA

Paired-end Illumina 75-bp sequencing files were mapped to the mouse genome GRCm38/mm10 database using the Bowtie266 alignment tool. Reads were quantified using Salmon72 and the count tables were analysed using DEseq273 (version 1.18.1), using Wald statistics(Log2 fold change > 1, padj value < 0.001) for pairwise comparison and Likelihood Ratio Test statistics (FDR < 0.01) to identify statistically different expression patterns across timepoints. Gene Ontology enrichment analysis was performed with clusterProfiler74 (version 3.6.0). Anatomy ontology enrichment was performed MouseMine web interface71.

ChIP-seq analyses
ChIP data for undifferentiated mESCs were generated previously15. All fastq files were mapped using bwa (version 0.7.10-r789) and filtered using samtools (version 1.7, flag -F 1024), then normalized for depth of sequencing. Peak-calling was done using MACS2-2.775 (qvalue <0.0001). Only peaks that were called in both replicates were used downstream. Heatmaps for ChIP-seq were generated using fluff76(v3.0.2) from bam files using read-deapth normalization. Reads Per Kilobase of transcript, per Million mapped reads (RPKM) quantification from two independent replicates were performed using scipy (v 1.1.0). GimmeMaelstrom77 (v 0.14.0) was used for Fig. 5c to identify the transcription factor motifs that are influencing RNA expression dynamics by scanning motifs associated with promoters (+/- 0.5kb from TSS) of a list of differentially regulated genes for both Mtf2 and Jarid2 null cells against their own wild-types at the undifferentiated stage. By default GimmeMaelstrom uses a non-redundant, clustered database of known vertebrate motifs: gimme.vertebrate.v5.0. These motifs come from CIS-BP (http://cisbp.ccbr.utoronto.ca/) and other sources such as JASPAR, IMAGE, HOMER and Swiss Regulon. DNA shape analysis was performed using the DNAshape package78.

For manuscripts utilizing custom algorithms or software that are central to the research but not yet described in published literature, software must be made available to editors and reviewers. We strongly encourage code deposition in a community repository (e.g. GitHub). See the Nature Research guidelines for submitting code & software for further information.

Data

Policy information about availability of data

All manuscripts must include a data availability statement. This statement should provide the following information, where applicable:

- Accession codes, unique identifiers, or web links for publicly available datasets
- A list of figures that have associated raw data
- A description of any restrictions on data availability

The Single-cell RNAseq and bulk RNAseq fastq and count matrices have been deposited in the GEO repository under accession code GSE154572 (https://www.ncbi.nlm.nih.gov/geo/query/acc.cgi?acc=GSE154572). ChIP-seq reads, coverage as genome browser tracks, and peak files have been deposited in the GEO repository under accession code GSE94300. The single cell data can be downloaded and viewed in a user interface via the following repository - https://github.com/chethloh/PRC2_singlecelldata

Field-specific reporting

Please select the one below that is the best fit for your research. If you are not sure, read the appropriate sections before making your selection.

☒ Life sciences ☐ Behavioural & social sciences ☐ Ecological, evolutionary & environmental sciences

For a reference copy of the document with all sections, see nature.com/documents/nr-reporting-summary-flat.pdf

Life sciences study design

All studies must disclose on these points even when the disclosure is negative.

Sample size	For Single-cell studies, we aimed for at least 1200cells per experimental condition to have 95% confidence that our sample will contain at least 5 cells from each of the assumed cell types (10 cell types), and that they can be present at a fraction of 1% of the total population (https://satijalab.org/howmanycells/). Bulk-RNA seq was conducted with 2 technical replicates per condition. Single-Cell RNA seq was performed on cells derived from pooled 6-well plates of embryoid bodies from different timepoints and conditions. ChIP-seq was performed in two independent replicates, as recommended by the ENCODE ChIP-seq guidelines.
Data exclusions	No data was excluded.
Replication	All ChIPseq and bulk RNA-seq experiments were performed in duplicates. Replicates passing QC were considered successful replication of the experiments. Individual replicates were consistent for the effects reported. Directed differentiation of ESCs were also conducted with 2 technical duplicates per condition. and differentiated were repeated with success for at least two different occasions.
Randomization	Complete randomization is not applicable to our differentiation experiments deisgn. Cells of known genetic backgrounds were compared against their respective wild-type controls, and all treatments were the same between samples.
Blinding	Clustering and Pseudotime analyses of single-cell data were performed in an unbiased manner. Complete blinding is not applicable to bulk-rna seq and chip-seq experiments because of the nature of the validation experiments, like qpcr checks which require prior knowledge of which samples we were using to investigate specific gene sets that are associated with them.

Reporting for specific materials, systems and methods

We require information from authors about some types of materials, experimental systems and methods used in many studies. Here, indicate whether each material, system or method listed is relevant to your study. If you are not sure if a list item applies to your research, read the appropriate section before selecting a response.

Materials & experimental systems

n/a	Involved in the study
☐	☒ Antibodies
☐	☒ Eukaryotic cell lines
☒	☐ Palaeontology and archaeology
☒	☐ Animals and other organisms
☒	☐ Human research participants
☒	☐ Clinical data
☒	☐ Dual use research of concern

Methods

n/a	Involved in the study
☐	☒ ChIP-seq
☒	☐ Flow cytometry
☒	☐ MRI-based neuroimaging

Antibodies

Antibodies used	ChIP was performed using 3 µl per sample of the following antibodies: MTF2 (Aviva System Biology ARP34292, lot QC49692-42166), H3K27me3 (Millipore 07-449, lot 2717675), EZH2 (Diagenode C15410039, lot 003), H3K4me3 (Ab858, lot GR240214-4) and Anti-GATA-2 Antibody (H-6) (Santa Cruz, #sc-515178, 1:500).
Validation	All antibodies were validated for use in mouse and human cells by the manufacturers and are ChIP/IP grade.

Eukaryotic cell lines

Policy information about cell lines

Cell line source(s)	E14, Mtf2GT/GT ref49, Jarid2-/- ref7 and Eed-/- ref61, Mtf2WT(Pcl1-3wt) ref20, Eed WT(J1) ref61&63 and Jarid2WT(JM8) ref62. Wild type (WT) cells of Mtf2 null (Pcl1-3 wt ESCs ref 22), Jarid2 null (JM8 ESCs ref63) and Eed null (J1 ESCs refs 62 and 64). We used background-matched wild type (WT) cells of Mtf2 null (Pcl1-3 wt ESCs ref 22), Jarid2 null (JM8 ESCsref 63) and Eed null (J1 ESCs refs 62,64) for bulk RNA sequencing and ChIP analyses.
Authentication	Mtf2GT/GT were genotyped by PCR as in ref 49,Jarid2-/- and Eed-/- by WB
Mycoplasma contamination	All lines are routinely tested for mycoplasma contamination
Commonly misidentified lines (See ICLAC register)	No commonly misidentified cell lines were used

ChIP-seq

Data deposition

☒ Confirm that both raw and final processed data have been deposited in a public database such as GEO.

☒ Confirm that you have deposited or provided access to graph files (e.g. BED files) for the called peaks.

Data access links *May remain private before publication.*	ChIP-seq reads, coverage as genome browser tracks, and peak files have been deposited in the GEO repository under accession code GSE94300.
Files in database submission	List of all files can be located on GSE94300 database
Genome browser session (e.g. UCSC)	Data track hub for UCSC Genome Browser, http://veenstra.science.ru.nl/trackhubm.htm and http://trackhub.science.ru.nl/hubs/mouse_veenstra/hub.txt; spp package, https://github.com/hms-dbmi/spp.

Methodology

Replicates	ChIP-seq was performed in two independent replicates, as recommended by the ENCODE ChIP-seq guidelines.
Sequencing depth	42-bp paired end sequencing was performed. On average 38million QC-passed uniquely mapped reads per ChIP sample were derived.
Antibodies	ChIP was performed using 3ul/sample of the following antibodies: MTF2 (Aviva System Biology ARP34292, lot QC49692-42166), H3K27me3 (Millipore 07-449, lot 2717675), EZH2 (Diagenode C15410039, lot 003), H3K4me3 (Ab8580, lot GR240214-4), JARID2 (Novus Biologicals NB100-2214, Lot E2) and GATA2(H-6, Santa Cruz, #sc-515178).
Peak calling parameters	Base-calling was performed by the Illumina CASAVA software. Reads were aligned to the mouse genome (mm10) using bwa 0.7.10-r789 with default settings. Peaks were called with MACS2-2.7(Zhang et al., 2008) using the --nomodel option and manual shift provided with the –extsize parameter.

Data quality

The extent of peak shifting was calculated with spp R library (https://github.com/hms-dbmi/spp). A q value threshold of 0.001 was applied in all cases and either the --call-summits (MTF2 and EZH2) or the --broad (H3K27me3 and H3K4me3) parameters was used. High-confidence conserved peaks were identified with MAnorm(Shao et al., 2012) allowing a maximum of 1.5 fold change between replicates. Peak summits were defined as the nonoverlapping 100bp region around the summits called by MACS in the high confidence peaks

Software

Heatmaps of ChIPseq signal were generated using fluff v2.1.0(Georgiou and van Heeringen, 2016). The same analysis pipeline was applied for analysis of published data. Motif search and kmer analysis was performed with GimmeMotif v0.8.6(van Heeringen and Veenstra, 2011) and kmer-SVM v1.0(Lee et al., 2011). Reads Per Kilobase of transcript, per Million mapped reads (RPKM) quantification from two independent replicates were performed using scipy (v 1.1.0).
